# Cost-effectiveness of collaborative care including PST and an antidepressant treatment algorithm for the treatment of major depressive disorder in primary care; a randomised clinical trial

**DOI:** 10.1186/1472-6963-7-34

**Published:** 2007-03-01

**Authors:** Marjoliek A IJff, Klaas ML Huijbregts, Harm WJ van Marwijk, Aartjan TF Beekman, Leona Hakkaart-van Roijen, Frans F Rutten, Jürgen Unützer, Christina M van der Feltz-Cornelis

**Affiliations:** 1 Program Diagnosis and Treatment, Netherlands Institute of Mental Health and Addiction, Utrecht, The Netherlands; 2 Institute for Research in Extramural Medicine, VU Medical Centre, Amsterdam, The Netherlands; 3 Department of General Practice, VU Medical Centre, Amsterdam, The Netherlands; 4 Department of Psychiatry, VU Medical Centre, Amsterdam, The Netherlands; 5 Institute for Medical Technology Assessment, Erasmus Medical Centre, Rotterdam, The Netherlands; 6 Center for Health Services Research, UCLA Neuropsychiatric Institute, Los Angeles, California, USA

## Abstract

**Background:**

Depressive disorder is currently one of the most burdensome disorders worldwide. Evidence-based treatments for depressive disorder are already available, but these are used insufficiently, and with less positive results than possible. Earlier research in the USA has shown good results in the treatment of depressive disorder based on a collaborative care approach with Problem Solving Treatment and an antidepressant treatment algorithm, and research in the UK has also shown good results with Problem Solving Treatment. These treatment strategies may also work very well in the Netherlands too, even though health care systems differ between countries.

**Methods/design:**

This study is a two-armed randomised clinical trial, with randomization on patient-level. The aim of the trial is to evaluate the treatment of depressive disorder in primary care in the Netherlands by means of an adapted collaborative care framework, including contracting and adherence-improving strategies, combined with Problem Solving Treatment and antidepressant medication according to a treatment algorithm. Forty general practices will be randomised to either the intervention group or the control group. Included will be patients who are diagnosed with moderate to severe depression, based on DSM-IV criteria, and stratified according to comorbid chronic physical illness. Patients in the intervention group will receive treatment based on the collaborative care approach, and patients in the control group will receive care as usual. Baseline measurements and follow up measures (3, 6, 9 and 12 months) are assessed using questionnaires and an interview. The primary outcome measure is severity of depressive symptoms, according to the PHQ9. Secondary outcome measures are remission as measured with the PHQ9 and the IDS-SR, and cost-effectiveness measured with the TiC-P, the EQ-5D and the SF-36.

**Discussion:**

In this study, an American model to enhance care for patients with a depressive disorder, the collaborative care model, will be evaluated for effectiveness in the primary care setting. If effective across the Atlantic and across different health care systems, it is also likely to be an effective strategy to implement in the treatment of major depressive disorder in the Netherlands.

## Background

The burden of depressive disorder on society and on individual patients is enormous. The global burden of disease study reports that unipolar major depressive disorder is expected to be one of the top 2 leading causes of disability-adjusted life years in 2020 [1]. In primary care, 10% of the patients have a depressive disorder -although they may not explicitly seek help for this condition- [2], and when taken together with comorbid anxiety disorders, the community prevalence increases to an alarming 15–20% [2]. Moreover, up to 30% of the population will suffer once in their lifetime from depressive or anxiety symptoms [2,3]. Depressive disorder has major consequences for the lives of patients, but is treatable. However, it is frequently under-diagnosed and under-treated in primary care [4]. Hence there is clearly room for improvement in the diagnosis and treatment of depressive disorder in primary care.

When looking at pitfalls in the efficient diagnosis and treatment of depressive disorder in primary care, diagnosis is paramount for the success of the treatment. However, several barriers hamper the diagnostic process. Patients often present themselves with several problems at the same time, and the general practitioner (GP) has to deal with 'competing demands', as described by Nutting [5]. In primary care a patient-led agenda is usually followed, and this reduces the chance of detection, because many depressed patients tend to avoid acknowledgement of their distress [5]. Moreover, a European study has shown that the length of the consultation increases drastically when psychosocial problems are detected by the GP [6]. Therefore, GPs do not always focus on the possibility of an underlying mental disorder such as depression.

Detection is further impaired, because 70% of depressed patients present themselves with physical symptoms and not with depressive symptoms [7]. In addition, the comorbidity between somatoform disorders and depression is high; earlier research shows a prevalence of 16.1% for somatoform disorders in primary care, and a 3.3 times greater likelihood of comorbid anxiety/depressive disorder than can be expected by chance [8]. Medically unexplained symptoms are thus, even though not by rule, at least a possible symptom of depressive disorder, and should therefore be addressed. Comorbid somatoform disorder influences the outcome of treatment; comorbid pain with depression, for example, predicts a longer time to remission [9].

Depressive disorder with comorbid physical chronic illness is highly prevalent in primary care [10-13] and it negatively influences (treatment of) the comorbid physical illness, and vice versa [14,15]. For instance, the course of major depressive disorder in patients with diabetes tends to be more severe, with recurrences being the norm rather than the exception. However, even if these patients are treated successfully, approximately 80% will experience a recurrence [14]. Comorbid depressive disorder is associated with a lower quality of life [16,17] poor adherence to (diabetic) treatment [14,18] and also increased health care costs [19,20]. Therefore, in the present study attention will be paid to comorbid somatoform disorder and physical chronic illness as factors in the accurate diagnosis and treatment of depressive disorder.

In primary care, evidence-based treatments for depressive disorder are available, but these are used insufficiently, and with miscellaneous results [4,21]. Impeding factors are non-acceptance of the diagnosis, non-adherence of patients to the treatment, and non-adherence of GPs [4]. Earlier research has shown that interventions developed in the mental health setting can also be effective in primary care [22], but screening per se [23] and simple interventions [24] alone are not effective. Many single treatment modes -for instance, pharmacotherapy, psychotherapy or GP training- have been tried out in primary care, but with disappointing results; the treatment modes are, in fact efficacious, but their effect is limited [23]. The efficacy of a specific treatment mode is usually studied in a controlled clinical trial, but the implementation in daily practice under uncontrolled conditions can (and does) lead to different and sometimes worse outcomes [23,25]. Schulberg therefore opts for ways to transfer efficacious treatments from research settings to routine primary care in a way that 'permits them to flourish', recommending various steps to execute this transfer effectively [25]. Multi-faceted, patient-tailored methods of treatment are needed, in combination with improvement of adherence, i.e. disease-management programmes such as collaborative care [24,26-29]. Two recent meta-analyses have indeed shown the effectiveness of such collaborative care approaches in primary care [30,31].

The collaborative care framework used in the present study is based on enhancing active collaboration of the patient with the providers of the treatment, and active collaboration between primary care and mental health care [26,32]. Within this collaborative care framework the following interventions are implemented: care-management [32,33], contracting [29] (the care manager explains the nature of the symptoms as part of a depressive disorder, mentions several evidence and guideline-based treatment options, and formulates a treatment plan, together with the patient and the GP in accordance with the preferences of the patient), adherence-enhancing techniques, manual-guided self-help and/or lifestyle changes, Problem Solving Treatment (PST) [34-36] and antidepressant medication according to a treatment algorithm. Psychiatric consultation is one of the adherence-enhancing techniques in the stepped care protocol. Depending on the patients preferences in the contracting phase, the collaborative care treatment plan contains (one of) these modules. Contracting and self help are novel within this collaborative care approach. Contracting is included because it has been proven to be an effective way to enhance adherence [29], and self-help because this strategy has a long history of positive results in the Netherlands [37-40]. In Wagner's chronic care model, self-help has always been a major aspect of good chronic illness care, and in the IMPACT study [27,32] self-help was also included and referred to as education for self management. In the present study this aspect is emphasized even more. This will be described in more detail in another publication [41].

One of the elements in the collaborative care treatment model is a type of evidence-based psychotherapy for depression that is feasible in primary care. An option that meets these requirements is PST, a brief psychological intervention that is based on problem solving therapy from the behavioural perspective, but is less time-consuming [36,42]. Research in the UK has recently shown that PST provided by suitably trained practice nurses is effective in the treatment of depressive disorder in primary care patients [34,35]. The IMPACT study in the USA also successfully implemented PST within a collaborative care approach in the treatment of depressive disorder in primary care patients [32].

Algorithms describing the stepped use of antidepressant medication already exist, but are mainly used in secondary care [43-45]. Antidepressant medication is effective in the treatment of major depressive disorder in primary care [46], but current practice can be further improved by adherence-enhancing strategies according to an algorithm that has been specifically developed for the primary care setting. Elements of the present treatment algorithm are specific decision points for stepped care and recommendations for the choice of medication and daily dosages, implemented by the GP and reinforced by a consultant psychiatrist.

The aim of the current randomised clinical trial (RCT) is to study the effectiveness of treatment for depressive disorder in primary care in the Netherlands based on a collaborative care framework, and including care management, contracting, adherence-improving strategies, manual-guided self help and lifestyle interventions, PST, and an antidepressant treatment algorithm, in which the treatment plan is based on patient preferences, and compared with well-documented care as usual (CAU) as provided by the GP.

The interventions included in the collaborative care package are all evidence-based, and the trial will test the whole package for effectiveness in primary care (compared to care as usual). This study is therefore a pragmatic trial, not an efficacy trial, because it is not the intention to evaluate new interventions [47]. Economic analyses will be performed to assess whether collaborative care is not only effective in terms of response and remission to treatment, but also effective in terms of (health care) costs.

## Methods/design

### Objectives

The primary aim of the current study is to evaluate the effectiveness of a collaborative care approach, including contracting, PST and an antidepressant treatment algorithm in the treatment of major depressive disorder in primary care. Secondary aims are to assess remission, and to estimate the cost-effectiveness of the intervention.

### Study design

The study design is a two-armed randomised pragmatic clinical trial, stratified according to physical chronic illness.

**Figure 1:**
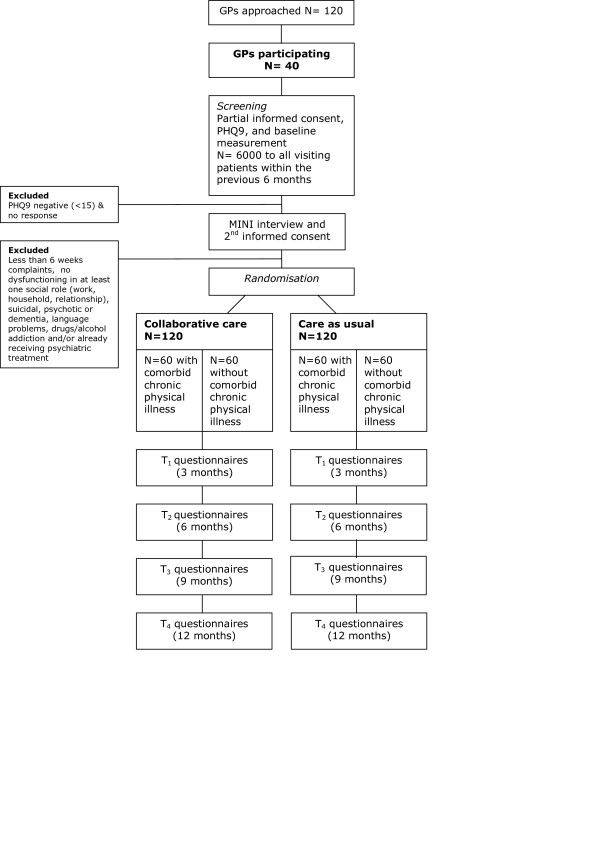
**Flowchart of participants**. GP: general practitioner, PHQ9: Patient Health Questionnaire depression sub-scale, MINI interview: Mini-International Neuropsychiatric Interview

Subjects are randomised and allocated to the intervention (collaborative care) or the control group (CAU). See Figure 1 for flowchart of participants.

### Recruitment of GPs

The study is designed in cooperation with the Department of General Practice at the VU Medical Centre in Amsterdam. From the group of general practices connected to this department, as well as from general practices connected to a regional support structure for primary care in two other regions in the Netherlands, practices will be recruited.

### Recruitment of patients

The aim is to include patients with a diagnosis of major depressive disorder and who dysfunction due to this depressive disorder (i.e. by the loss of role-functioning in daily life).

All patients who visited the participating practices within the previous 6 months will be selected from the files. These patients will receive a letter describing the purpose of the study, together with an informed consent form for the screening procedure, the Patient Health Questionnaire depression sub-scale (PHQ9) [48] that is used as a screener for depressive disorder, and baseline questionnaires. Any other patients that the GPs recommend for participation will also receive the informed consent letter, the PHQ9 and the baseline questionnaires.

Patients will be included if they reach the cut-off score of 15 for moderate to severe depressive disorder on the PHQ9 and also give informed consent [48]. Subsequently, the Mini-International Neuropsychiatric Interview (MINI [49,50] will be administered for the classification of symptoms and the diagnosis of major depressive disorder. In the same interview the patients will be asked if they have had the symptoms for at least 6 weeks, and if their dysfunctioning leads to serious problems in at least one of the following roles: work, household activities or relationship.

In the intervention group, the patients will be invited for a consultation with their GP; in the control group, the patients will receive CAU.

### Patient exclusion criteria

Patients will be excluded from the study if they are suicidal, psychotic, or suffering from dementia, have insufficient knowledge of the Dutch language to fill in the questionnaires, are addicted to drugs or alcohol, are already receiving psychiatric treatment and/or are under 18 years of age.

### Intervention

#### Training

Since the treatment includes a collaborative care intervention, the participating GPs and care managers will receive training in collaborative care, including contracting and the antidepressant treatment algorithm. The practice nurses will also be trained in care management and will be trained in PST.

The training will be given by the research group. All members of the research group had received training from the IMPACT research group in Seattle [32], the developers of the collaborative care model.

#### Treatment in the intervention group

##### 1. Collaborative care

The care provided in the intervention group follows a collaborative care approach: patient-tailored care executed, in the current study, within a team of the GP, the patient, the care manager and the consultant psychiatrist. Within this team the need for and the specific requirements for treatment are assessed, and a treatment plan is developed by the GP, the care manager and the patient. Subsequently, the care manager coordinates the care and evaluates each step. If necessary, each treatment step is followed up by a next step to improve the outcome according to the principles of stepped care [51]. The care manager consults the psychiatrist at set points in time, and if there are any difficulties. Subsequently, the care manager advises the GP when necessary.

##### 2. Contracting

During the initial visit, the care manager informs the patient about depressive disorder, and discusses treatment options with the patient. The patient is then offered the choice between several types of treatment (PST and antidepressant medication if desired) and, according to the patient's preferences, a treatment plan is jointly formulated by the care manager, the patient and the GP together.

Initially, the progress of the patient according to the treatment plan is monitored by the care manager, weekly or fortnightly, by telephone or during visits to the clinic. The PHQ9 is filled in, and the results are discussed. When the symptoms are in remission, a relapse prevention plan is formulated by the care manager and the patient together (e.g. defining alarming symptoms and maintenance of antidepressant medication) and follow-up becomes less frequent.

If the patient does not respond (or responds only partially) to the initial treatment, and/or if a patient is not satisfied with the chosen treatment, another form of treatment will be contracted. Subsequent steps are again based on the patient's preferences, and follow the principle of stepped care supervised by the consultant psychiatrist.

##### 3. Improving adherence

The adherence of GPs has been found to be enhanced by a combination of phone calls, written instructions and reminders; an approach that is feasible and effective [24,27,52]. The care manager is likewise closely monitored via a net-based tracking system, with set evaluation points every six weeks under the supervision of the consultant psychiatrist. There will be PST supervision, and monthly meetings with other care managers will be held to improve performance as well as adherence.

Patient adherence is improved by contracting and providing written instructions for the patient [24,53] and the care manager, who monitors the patient meticulously [32].

##### 4. Manual-guided self-help

One of the core symptoms of depression is the loss of interest in (nearly) all activities during the best part of the day, almost every day [54]. In order to relieve these symptoms, a self-help manual has been developed, activating the patients step-by-step as they read each chapter. Initially, the patients are encouraged to participate in low-level behavioural activation, depending on their capability. Attention is paid to restoring the sleep-wake-rhythm [55], self-relaxation-techniques [37,56] and diet [40,57]. As the patient improves, there are options for running-therapy, [37,58] which has recently been proved to be helpful in the treatment of (minor) depressive disorder, and promoting return to work. Two-thirds of the costs involved in the treatment of depressive disorder are accounted for by absence from work through illness, as Bosmans recently pointed out [59].

##### 5. Treatment algorithm for antidepressant medication

To enhance the effectiveness of and adherence to antidepressant medication, the GPs are provided with a protocol of instructions to optimize the prescription of antidepressants. The protocol includes time to titrate to daily dosages (2 weeks), time to respond (partially or remission, another 4 weeks), and well-defined step up criteria and methods (for example 'partial response: increase dose', 'no response: switch medication'). Progress (i.e. (partial) response) is measured with the PHQ9, and results and adverse effects are discussed with the care manager. Subsequently, the care manager advises the GP to step up the treatment (if necessary, after consulting a psychiatrist) if the results are not satisfactory and/or adverse effects are intolerable.

##### 6. Problem solving treatment

PST is provided by the care manager in a course of 6–12 sessions, depending on the progress on the PHQ9 scale. The problem solving approach consists of 7 stages, and is based on the concept that emotional problems are often induced by problems in daily life [35]. The focus lies on regaining control over symptoms by doing something about these problems. The first session lasts for one hour, during which, based on the patient's current problems a clear focus of the treatment is established. Follow-up sessions last for 30 minutes.

Stepped care is realized by evaluation points at 6 weeks and 12 weeks at which it is decided if one or two antidepressants, and 6 sessions or 12 sessions of PST will be sufficient treatment, depending on the improvement according to the PHQ9. It is possible to switch between the two options (e.g. due to disappointing results, or adverse effects) or, if the patients prefers this, to receive a combination of the two treatment options. Either way, the total intervention will last for 18 weeks. This is the maximum duration of the studied intervention.

#### Treatment in the control group

Half of the general practices function as a control group, and in these practices the patients receive CAU. The actual content of the CAU treatment (e.g. medication, number of contacts in primary care, and referral policy) will be assessed with the Scale for Medical Utilisation of Health Services [53].

### Outcome-parameters

#### 1. Primary outcome measure

##### Response

The severity of the depressive symptoms is measured with the Patient Health Questionnaire depression sub-scale, [48] a brief but valid instrument that scores each of the DSM-IV criteria for major depressive disorder. Response is defined as a 50% reduction of symptoms [48].

#### 2. Secondary outcome measures

##### Remission of depressive symptoms

Remission -reduction of DSM-IV criteria below the threshold for a diagnosis of depressive disorder- [54] is measured with the PHQ9 [48]. As in previous studies [32,48,60], remission is defined as a score between 0 and 4 on the PHQ9.

##### Cost utility

In addition to the improvement in the severity of symptoms, the cost utility of collaborative care compared to CAU is also assessed. Therefore, the direct medical costs and the costs due to production losses (productivity costs) will be estimated. The relevant data will be collected by means of the Trimbos/iMTA questionnaire for Costs associated with Psychiatric Illness (TiC-P) [61,62]. Quality of life is assessed with the EuroQol (EQ-5D) [63] and the Short Form-36 (SF-36) [64], which are validated tools for measuring general health-related quality of life.

The EQ-5D descriptive system consists of five dimensions (Mobility, Self Care, Usual Activities, Pain/Discomfort and Anxiety/Depression) each with three levels (no problems, some problems and extreme problems), thus defining 243 (3^5^) distinct health states. Recently, a study in the Netherlands measured and valuated the EQ-5D in a national setting, resulting in the 'Dutch EQ-5D tariff'. The resulting tariff is used to calculate utilities for EQ-5D health states for cost-utility analyses ofDutchhealth care programmes and treatments.

The cost utility is evaluated by relating the difference in direct medical costs per patient receiving collaborative care or CAU to the difference in terms of Quality Adjusted Life Years gained (QALY), which yields a cost per QALY estimate. Furthermore, an attempt will be made to estimate the cost per QALY, including the productivity costs.

#### 3. Effect modifiers and additional outcome measures

Effect of physical illness. Physical comorbidity is measured by means of a questionnaire developed by the Dutch Central Bureau for Statistics (the CBS list). The list contains 28 chronic conditions (e.g. diabetes type II and Multiple Sclerosis). Physical illness is thought to be the key effect modifier.

Somatoform disorder, as measured with the Lichamelijke Klachten Vragenlijst: Bodily Complaints Questionnaire (LKV; assessing the number and intensity of functional somatic complaints a patient is experiencing) [65], the Screening for Somatoform Symptoms-7 (SOMS-7; measuring possible comorbid somatoform disorder) [66], the Illness Attitude Scale (IAS; measuring concern about disease) [67] and the Whitely Index (measuring Hypochondriasis) [67].

Concomitant symptoms of comorbid chronic illness, measured with the Multidimensional Fatigue Inventory (MFI) [68,69], and the World Health Organization Disability Assessment Survey II (WHO-DAS-II) [70].

Depressive symptoms are assessed as well with the 30-item Inventory for Depressive Symptomatology Self-Report (IDS-SR) [71]. The IDS-SR measures all DSM-IV criteria for the diagnosis of major depressive disorder, as well as the symptoms that are most frequently associated with major depressive disorder (e.g. melancholic symptoms and anxiety) [72].

Preference and adherence. Possible patient preference is based on the preferences of patients in the intervention group, patient adherence is assessed with a qualitative questionnaire, and the patient-doctor working relationship (from the perspective of the patient) is assessed with the Patient Doctor Relationship Questionnaire (PDRQ-9) [73]. The attitude of the GP towards the treatment of depressive disorder is measured with the Depression Attitude Questionnaire (DAQ) [74].

Life-events and social support are measured according to the Social Readjustment Rating Scale [75] and two items of the SF-36 [64], respectively.

Personality is assessed with the Neuroticism extraversion openness-five factor inventory (NEO-FFI; sub-scales 'extraversion' and 'neuroticism') [76,77].

Treatment in the CAU group is assessed with the Contact between Patients and Practitioners Questionnaire (filled in by the patient) and the Questionnaire Medical Consumption (filled in by the GP) [53].

Baseline measurements take place before inclusion (T_0_), and follow-up measurements take place after 3 months (T_1_), 6 months (T_2_), 9 months (T_3_) and 12 months (T_4_). 

### Sample size

The primary outcome measure is response (a 50% reduction in depressive symptoms). Based on previous work [27] the expected response rate is 14,76% in the CAU group and 31,8% in the intervention group. Given these estimates of the effect of the intervention, and using conventional values for α (0.05) and β (0.80), and two-tailed tests with equal groups, the sample size aimed for in the current study is 94 patients per arm. To compensate for an estimated 26% loss to follow up, 120 patients per arm will be included. (See Figure 1 for flowchart participants.

### Analysis

Intention-to-treat analysis will be performed with t-tests, Chi-square tests (dichotomous variables), and General Linear Model (GLM) repeated measurements (continuous variables). The effect size will be estimated by Chi-square tests, and described in Cohen's d. Time to remission will be analysed using survival analysis, and Cox regression models for multivariate analyses will be used for possible effect modifiers (e.g. physical chronic illness and somatoform disorder). Possible confounders such as age, gender, immigrant status, level of education, history of treatment and life events will also be included as variables in these analyses.

### Economic evaluation

The aim of the economic evaluation is to assess the cost-effectiveness of collaborative care of the treatment of depressive disorders in primary care. A cost-utility analysis (CUA) will be applied, and the results will be expressed as cost per QALY. The economic evaluation will be undertaken from a societal perspective. Hence, all relevant effects and costs due to resource utilisation within the health care system (direct medical costs) and costs due to production losses (productivity costs) will be included.

Since the collaborative care intervention used in this study is a new intervention, a unit price per session is not known yet. To determine a reference price, a detailed cost-price study will be performed. Therefore, we will perform measurements of time for face-to-face contacts as well as indirect time per contact (e.g. consultations of other specialists) for a total of 20 sessions. Furthermore, we will estimate overhead costs based on the information of the financial department of the hospital. This will result in an estimate of the actual costs per contact. The unit cost estimate per contact will be used as a reference price per contact for the collaborative care intervention.

The TiC-P [61] will be used to assess the costs. The TiC-P is commonly applied in the economic evaluation of treatment in mental health care. For instance, the TiC-P was recently used in a large naturalistic trial on the cost utility of brief psychological treatment for depression and anxiety [62].

To calculate the total direct medical costs, the total number of medical contacts (outpatient visits, length of stay in hospital, use of medication, etc will be multiplied by unit costs of the corresponding health care services. Reference unit prices of health care services will be applied, and adjusted to the year of this study according to the consumer price index [78].

In the second part of the TiC-P a short form of the Health and Labour questionnaire (HLQ) will be used to collect data on productivity losses [79]. The Short-Form HLQ (SF-HLQ) consists of three modules that measure productivity losses: absence from work, reduced efficiency at work and difficulties with job performance [80]. The number of days absent from work and the actual cost of hours missed at work due to health-related problems are calculated from the average value added per worker according to age and gender per day and per hour, respectively. If respondents indicate that they have been absent from work for the entire recall period, data will be collected as from the date when the period of long-term absence started. This additional information will be used to calculate the production losses according to the friction cost method [81,82]. The friction cost method takes into account the economic circumstances that limit the losses of productivity to society, which is related to the fact that a formerly unemployed person may replace a person who becomes disabled [81].

For the economic evaluation, the effects will be measured by calculating utility scores. In addition to the clinical outcome parameters, utility scores will supply additional information about the impact of collaborative care on the treatment of depressive disorder, compared to CAU on the general health-related quality of life. Furthermore, the results can be compared to a broad range of other health care interventions, also outside the mental health care setting.

The cost utility will be evaluated by relating the difference in direct medical costs per patient receiving collaborative care or CAU to the difference in terms of QALYs gained, which yields a cost per QALY estimate. Furthermore, the cost per QALY, including the productivity costs, will be estimated.

In the case of missing data on costs and/or effects, and the additional uncertainty this introduces, multiple imputation will be used [83]. The Monte Carlo Markov Chain (MCMC) approach will be used to impute the missing values. The uncertainty will be assessed using bootstrapping, and the results will be presented in acceptability curves [84].

### Time-frame of the study

The preparatory period is 6 months. Subsequent to the approval of the Medical Ethical Board, general practices will be recruited and the GPs and care managers will be trained. The inclusion and intervention phase will take 22 months. The follow-up phase will be 6 months, and the data-analyses will last for 6 months. The entire study period will last for 4 years.

### Ethical principles

The study has been designed, and will be executed in accordance with the principles laid down in the Helsinki Declaration (Edinburgh, Scotland amendment, October 2000).

Participation in the study is voluntary, and written informed consent will be obtained. The patients will be explicitly informed of the fact that they can withdraw their consent to participate at any time, without specification of reasons and with no negative consequences with regard to their future medical treatment. Patients who wish to withdraw from the study will receive care as usual.

The study protocol has been approved by the Medical Ethics Committee of the VU Medical Centre.

## Discussion

The aim of this study is to investigate the effectiveness of an American model of collaborative care for the treatment of depression, implemented in the Netherlands. The emphasis lies on collaboration with a care manager, team effort and stepped care. Trials on such disease management programmes have so far shown promising results in the USA and the UK respectively [23,33,85-87], and are also likely to show the same results in the Netherlands. The focus hereby lies on major depressive disorder, since disease management programmes are developed to treat chronic diseases [32,88], and major depressive disorder closely resembles a chronic disease in terms of difficulty to treat, impact on daily life, duration, and chance of relapse [2].

### Strengths and limitations

Critics may raise concern about whether collaborative care -as designed in the USA- can be effectively implemented in the Netherlands, since the health care systems differ between the two countries. The American health care system is based on the comparatively rare private enterprise health care system (i.e. decentralized payment) whereas in the Netherlands there is a public insurance system (i.e. centralized payment). Primary care in the Netherlands is, in the majority of cases, provided in very small practices (1–2 GPs), and is in many ways less organized and less centralized than thelarger group practices in the USA. This could present a challenge in the implementation of a team-based approach with a care manager, because it is not likely that a solo general practice can really afford to have a full-time care manager, so the care managers will probably also have several other practice tasks. However, the collaborative care approach has been implemented in several settings, with different health care providers and different levels of CAU [32], and in each setting significant improvement has been reported for the collaborative care approach compared to CAU [27,30,31,89]. One can therefore expect to find the same improvement when the collaborative care approach is carefully implemented in the Netherlands.

A possible limitation of the study might also be that we are adding two elements, i.e. self-care (a self-help manual has been developed for this trial, enhancing patient empowerment) and contracting, in which the weighing of patient preferences for a given treatment is crucial. This might make the intervention stronger, but it might also make implementation more strenuous. Therefore, specific emphasis will be laid on adherence to these two additional aspects of the model.

Due to randomisation at patient level, one can argue that contamination of effect could occur: GPs and care managers might transfer their knowledge of the intervention to the control group. The risk of dilution of effect however, is expected to be very small. The positive effect of the intervention can only be reached by implementing the whole package, including the care manager who plays the most significant role within the model, and patients receiving care as usual are not involved with a care manager. The antidepressant treatment algorithm will be carried out by the GPs and not the care manager, but under strict supervision of the consultant psychiatrist, so dilution of effect in the control group, when medication is concerned, is also expected to be small.

A characteristic of collaborative care is the fact that the offered treatments are all evidence-based; efficacy thus has previously been proved. Effectiveness trials and reviews have already shown mixed but positive results concerning the optional treatment modes: short-term psychotherapy and antidepressant medication if desired. Either no significant difference is found between the effectiveness of antidepressant medication and psychotherapy [90-92] or, a combination of antidepressant medication and some form of psychotherapy is most effective [91,93]. A strong aspect of this design is the fact that we are offering both evidence-based treatment modes in a package.

Strengths and limitations taken into account, at the very least patients make a significant contribution to (the choice of) the treatment in this design. Aiming, above all else, to relieve patients of the burden of depressive disorder, this seems a particularly valuable contribution to the design.

## Competing interests

The author(s) declare that they have no competing interests.

## Authors' contributions

CFC is principle investigator, daily supervisor and consultant psychiatrist in the trial. HvM is daily mentor and AB is monthly supervisor. LHR will be supervising the cost-effectiveness part of the trial and FR contributed to the design. JU is principle investigator of the IMPACT trial in the USA and international advisor on the design. KH will be executing the trial together with MIJ. All participants have contributed their part of expertise and read and approved the latest version of the article.

## Pre-publication history

The pre-publication history for this paper can be accessed here:


